# Elective colectomy for treatment of benign colon polyps: National surgical trends, outcomes, and cost analysis

**DOI:** 10.1055/a-2689-5839

**Published:** 2025-10-16

**Authors:** Tonya Kaltenbach, Luke Martin, Jessica Yu, Benjamin Brooke, William Peche, Roy Soetikno, Mary Whooley, Andrew J Gawron

**Affiliations:** 1 VA San Francisco, University of California, San Francisco, San Francisco, United States; 2 14434 The University of Utah Health Sciences Center, Salt Lake City, United States; 3 20122 Specialty Care Center of Innovation, George E Wahlen Department of Veterans Affairs Medical Center, Salt Lake City, United States; 4 20122 Surgery, George E Wahlen Department of Veterans Affairs Medical Center, Salt Lake City, United States; 5 6684 Gastroenterology and Hepatology, Oregon Health Sciences University, Portland, United States; 6 6684 Medicine, Oregon Health & Science University, Portland, United States; 7 14434 University of Utah Health, Salt Lake City, United States; 8 19980 Gastroenterology, San Francisco VA Medical Center, San Francisco, United States; 9 8785 Medicine, University of California San Francisco, San Francisco, United States; 10 12348 Gastroenterology, University of Utah School of Medicine, Salt Lake City, United States; 11 20122 Gastroenterology, George E Wahlen Department of Veterans Affairs Medical Center, Salt Lake City, United States

**Keywords:** Endoscopy Lower GI Tract, Polyps / adenomas / ..., Endoscopic resection (polypectomy, ESD, EMRc, ...), Quality and logistical aspects, Quality management, GI surgery

## Abstract

**Background and study aims:**

Although endoscopic resection is recommended for management of complex benign colon polyps, patients are routinely referred for surgical resection. Little is known about the effects of these elective colectomies on patient outcomes. We sought to determine trends, surgical outcomes, and costs of elective colectomy for benign colon polyps.

**Patients and methods:**

We performed a retrospective cohort analysis of veterans nationwide using the National Veterans Affairs Surgical Quality Improvement Program (VASQIP) database linked to the national VA Corporate Data Warehouse database. We included all veterans (N = 7,102) undergoing elective colectomy for benign polyps from 2000 to 2015. Outcomes of interest were rates of colectomy, surgical pathology findings, morbidity, mortality, and costs.

**Results:**

Colectomy for benign polyps increased significantly from 6% of all colectomies in 2000 to 18% in 2014, and the percent of colectomies for colon cancer decreased from 40% to 31%. The 30-day mortality rate was 1.2% and the complication rate was 19.7%. Based on pathology, 80% of patients (n = 514) underwent right hemicolectomy, mean polyp size was 2.7 cm (± 1.7 cm), and 60.1% of resected polyps were adenomas. Median cost of colectomy was $22,712 for open and $20,697 for laparoscopic colectomy. Costs increased if a complication occurred.

**Conclusions:**

Rates of colectomy for benign adenomas significantly increased from 2000 to 2014. Colectomy was associated with significant mortality, morbidity, and cost. Development of strategies to improve endoscopic management of benign large colon neoplasms is urgently needed.

## Introduction

In the United States, colon cancer is the third most common cancer among both men and women 1 2 . Over the past three decades, incidence and mortality rates for colon cancer have decreased, largely attributed to higher rates of screening colonoscopy. Colonoscopy decreases mortality through earlier diagnosis of cancer and decreases incidence through polypectomy 3 4 5 . It is among the most commonly performed procedures (14 million/year) in the United States 5 .

Simple polypectomy is routinely performed at time of initial colonoscopy, whereas patients with more complex polypectomy may be referred for endoscopic resection by a specialist with experience in complex resection techniques, or for surgical removal 6 7 8 . Various advanced endoscopic resection techniques, such as endoscopic mucosal resection (EMR) or endoscopic submucosal dissection, have been established as safe and clinically effective in managing complex polyps 9 10 11 12 13 14 , and studies have highlighted the significant morbidity and mortality of colectomies for benign polyps 15 16 17 . Modeling studies have demonstrated endoscopic resection to be cost-effective compared with surgical resection 18 19 20 21 . In a US study over a 10-year period, surgical resection had significantly higher rates of adverse events (AEs) and also higher cost of Emergency Room visits after AEs 15 . However, a paucity of data on surgical outcomes following colectomy for non-cancerous polyps has limited conclusions regarding the optimal management strategy in this complex population. Furthermore, little is known regarding the actual colectomy utilization patterns for benign colorectal lesions in the United States, particularly in the Veteran Affairs Healthcare System (VA).

Understanding surgical practice patterns and outcomes for patients undergoing resection is crucial for making informed medical decisions, providing effective patient counseling, and establishing procedure-specific complication rates that can be used in future cost-effectiveness studies to determine the optimal management strategy. We hypothesized that rates of colectomies for benign polyps were increasing. We aimed to describe the trend, outcomes, and cost of elective colectomy for removal of benign colon polyps in a national cohort of patients who were veterans.

## Patients and methods

### Patient cohort and data sources

We identified all patients who were veterans and underwent colectomy for a benign colon polyp in the VA healthcare system between January 2000 and July 2015 using the Veterans Affairs Surgical Quality Improvement Program (VASQIP) database, a validated dataset containing preoperative, intraoperative, and postoperative patient characteristics 22 . We received approval from the University of California, San Francisco institutional review board (IRB), the San Francisco VA, the Salt Lake City VA Research and Development Committee, the University of Utah IRB, and the National VA Surgical Quality Data Use Group.

We queried the VASQIP database for colectomy procedures using Current Procedural Terminology (CPT) codes 44140, 44141, 44144, 44160, 44204, and 44205. Patients undergoing partial or complete rectal resections were not included. We identified two comparison cohorts based on primary indication using International Classification of Disease, version 9 (ICD-9) codes ( **Supplementary Table 1** ): colectomy for benign polyps and colectomy for colon cancer. Patients undergoing emergent operations were excluded. We then used the national VA Corporate Data Warehouse to assess patient characteristics, outcomes, and costs associated with each colectomy. We also selected a random subgroup of patients from this cohort for chart review of surgical pathology reports.

### Outcome measures

Primary endpoints were counts and trends over time of colectomy procedures. Secondary endpoints were procedure-related AEs, pathologic characteristics among the resected polyps as reported in the clinical pathology report, and costs.

For analysis of time trends, we included procedures completed during calendar years 2000 through 2014 because we only had partial data for the year 2015. We identified patients undergoing colectomy procedures for colon cancer (postoperative diagnosis code: ICD-9: 153.x, 154.x) from VASQIP as the reference group.

We assessed mortality after surgery at 30 days. Other AEs of interest included cardiovascular complications (myocardial infarction [MI], cardiac arrest, cerebrovascular accident, deep venous thrombosis), pulmonary complications (pneumonia, more than 48 hours of ventilator support, pulmonary embolism, reintubation), infectious/wound complications (sepsis, superficial skin and soft tissue infection [SSI], deep SSI, dehiscence) and urinary and renal complications (urinary tract infection [UTI], acute renal failure requiring dialysis, renal insufficiency not requiring dialysis). Time trends for complications were assessed with procedure years categorized in quartiles.

To examine polyp-related factors, pathology reports from a randomly selected subgroup were reviewed. Demographics, surgical outcomes, and time variations of the pathology review subgroup were compared with the main cohort. Pathologic characteristics of interest included specific resection type, number of polyps, polyp size, and histology.

Institutional and professional costs for hospitalization were reported as the total cost for a given hospitalization, including procedure costs. The cost associated with complications that occurred after initial hospital discharge was not included. Cost data were inflation-adjusted using the Personal Consumption Expenditures Index to 2009 dollars.

### Statistical analysis

Categorical variables were summarized as count (%), and continuous variables were summarized as mean and standard deviation (SD) or median and interquartile range (IQR). Normally distributed, continuous variables were compared with a *t* -test, wherease non-parametric comparisons were made with the Mann-Whitney U test. Chi-squared tests were used for analyses comparing the pathology sample to the larger cohort. Cuzick’s test was used to evaluate trends in procedures over time. Inflation-adjusted cost data are reported as median USD ($) and IQR. Median costs were compared with the Mann-Whitney U test.

## Results

### Cohort description

**Fig. 1:**
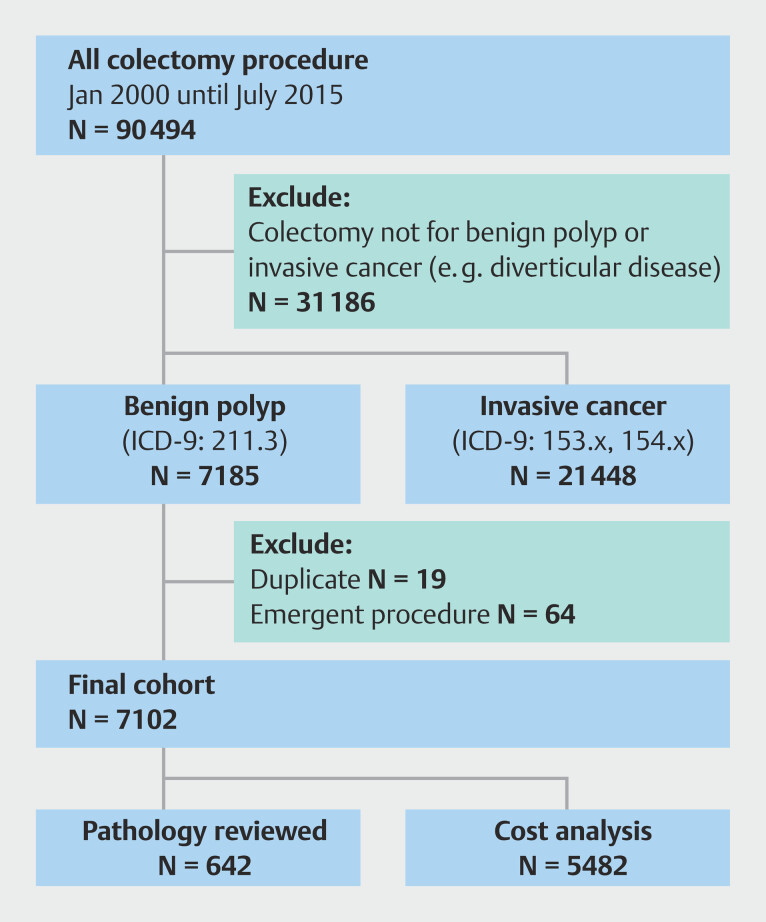
Study CONSORT diagram 23 .

We identified 60,494 patients who underwent colectomy from January 2000 until July 2015 ( Fig. 1 ). Of those, 21,448 were excluded for undergoing colectomy due to invasive cancer and 31,861 patients were excluded for having a colectomy for other indications. Finally, we excluded 19 duplicate patients and 64 emergent procedures. Our final cohort consisted of 7,102 veterans who underwent a colectomy procedure for a benign polyp during the study period ( Fig. 1 ). Patient demographics and preoperative characteristics are shown in Table 1 . Patients had a mean age of 66 years (SD 8.8). The majority were men (96.8%) and 70.7% of our patients were White. Patients had a mean body mass index of 28.7 (SD 4.7). The most common comorbidity was tobacco use (35.4%) followed by diabetes (25.4%). Most patients were American Society of Anesthesiologists class III (71.2%). Demographics of the pathology subgroup were not significantly different from the overall cohort.

**Table:** **Table 1** Descriptive patient demographic and preoperative characteristics.

**Patient characteristic**	**Elective colectomy for polyp** N = 7102 (%)	***P* value **
**Age, years** mean (SD)	66.0 (8.8)	0.88
**Race**		
White	4,530 (70.7)	0.97
Black	1,190 (18.6)	
Hispanic	254 (4.0)	
Other	434 (6.8)	
**Males**	6,873 (96.8)	0.59
** Body mass index, kg/m ^2^** Mean (SD)		0.61
**Comorbidities**		
Tobacco use	2,411 (34.0)	0.34
Alcohol use*	848 (12.0)	0.11
Diabetes†	1,803 (25.4)	0.45
Dyspnea‡	937 (13.3)	0.52
Congestive heart failure	60 (0.8)	0.12
Cerebrovascular accident	226 (3.2)	0.57
Preoperative weight loss§	183 (2.6)	0.36
Steroid use	96 (1.35)	0.64
Severe COPD	1,026 (14.5)	0.33
**ASA classification**		
I	17 (0.2)	0.52
II	1,595 (22.5)	
III	5,054 (71.2)	
IV	436 (6.14)	
Results are presented as number, percent or mean and SD as noted in the table. * ^1^ EtOH use defined as two or more drinks daily †Requiring medications including oral medications or insulin ‡Dyspnea at rest or with minimal exertion §Greater than 10% loss body weight in last 6 months. COPD, chronic obstructive pulmonary disease; EtOH, ethanol alcohol; SD, standard deviation.		

### Time trends for colectomy procedures

**Fig. 2:**
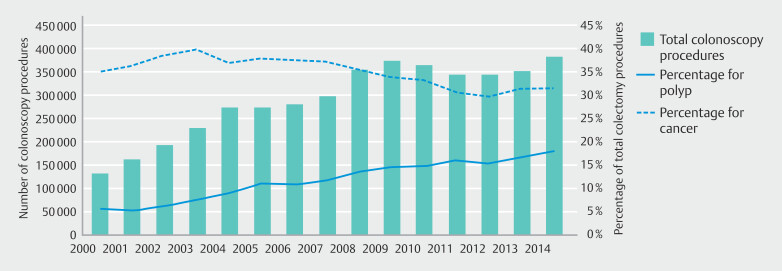
Comparison of aggregate colonoscopy procedures completed and percentage of colectomy cases completed by indication for surgery.

We found a significant increase in percentage of elective colectomy procedures done for benign polyps from 6% (n = 212) of the 3,825 colectomies performed in 2000 to 18% (n = 606) of the 3,367 colectomies performed in 2014 ( *P* < 0.001) ( Fig. 2 ). This increase in elective procedures for benign polyps paralleled observed increases in total colonoscopy procedures ( *P* < 0.001), which occurred rapidly between 2000 and 2008 ( Fig. 2 ). Initially, the yearly total number of colectomy procedures increased from 3,825 in 2000 until peaking in 2004 at 4,287, after which there was a steady and significant ( *P* < 0.001) downward trend. The initial increase was largely attributable to an increased number of procedures completed for colon cancer, which peaked in 2003 at 1,674 (40% of all colectomy procedures completed). The percentage of colectomy procedures completed for cancer trended downward thereafter ( *P* = 0.002).

### Surgical procedures and outcomes

Table 2 summarizes operative characteristics and postoperative complications. Median postoperative hospitalization length of stay was 6 days (IQR 5–8 days). Among all elective colectomy cases for benign polyp, 30-day mortality was 1.2%. The composite morbidity and mortality rate was 19.7%. The most common postoperative event was superficial SSI (7.6%, n = 538). In addition, 5.7% of patients (n = 384) required a reoperation, 2.7% of patients (n = 189) developed pneumonia, 2.7% of patients (n = 195) developed sepsis, and 2.6% of patients (n = 1 86) developed a UTI. Cardiac arrest occurred in 0.7% of patients (n = 51), and 0.1% of patients (n = 10) had a cerebrovascular accident. There were fewer UTIs (1.4%, n = 9) in the subgroup selected for pathology review, but postoperative complications in the subgroup were not statistically different from the entire cohort.

**Table:** **Table 2** Operative characteristics and surgical outcomes.

**Variable**	**Elective colectomy for polyp** N = 7,102 (%)	***P* value **
**Operative time (hours)** , mean (SD)	2.67 (1.13)	0.37
**Postoperative length of stay (days)** , mean (SD)	7.7 (7.0)	0.06
**Mortality** (30-day)	88 (1.2)	0.7
**Composite morbidity, mortality**	1,398 (19.7)	0.65
**Reoperation**	384 (5.4)	0.33
**Cardiac**		
Cardiac arrest	51 (0.7)	0.5
Cerebrovascular accident/stroke	10 (0.1)	0.32
Pulmonary embolism	40 (0.6)	0.37
Deep venous thrombosis	34 (0.5)	0.52
**Pulmonary**		
> 48 hours on vent	132 (1.9)	0.12
Pneumonia	195 (2.8)	0.73
Reintubation	170 (2.4)	0.58
**Urinary tract/renal**		
Acute renal failure	43 (0.6)	0.55
Renal insufficiency	62 (0.9)	0.48
Urinary tract infection	186 (2.6)	0.04
**Infection**		
Sepsis	189 (2.7)	0.31
Deep wound SSI	61 (0.9)	0.29
Superficial SSI	538 (7.6)	0.92
**Wound**		
Wound dehiscence	145 (2.0)	0.79
Results are presented as number, percent, or mean and SD as noted in the table. *Any one or more postoperative event. SD, standard deviation; SSI, superficial skin and soft tissue infection.		

### Time trends for surgical outcomes

Since 2000, laparoscopic colectomies increased, representing more than half of colectomies in 2014 ( **Supplementary Fig. 1** ). Mean operative time (hours) did not change (2.9 ± 1.4 [SD] in 2000 to 2.7 ± 1.1 [SD] in 2015, *P* = 0.347), although mean length of stay (days) decreased (9.7 ± 7.3 in 2000 to 6.8 ± 5.1 in 2015, *P* < 0.001). Although 30-day mortality rate and rate of any complication occurring after colectomies decreased ( **Supplementary Table 2** ), need for reoperation did not significantly change. Individually, only MI and deep soft tissue infection decreased; other specific postoperative AEs did not significantly change over the study period.

### Surgical pathology analysis

Surgical pathology characteristics from the subgroup are presented in **Supplementary Table 3** . Compared with the entire cohort, demographics of the subgroup were similar, but there were fewer postoperative UTIs and all but one procedure was performed after 2003 ( **Supplementary Table 3** ). Most procedures were performed on the right colon (ileocecectomy 4.1% and right hemicolectomy 80.1%) and fewer were completed on the left (sigmoid 5.0% and left hemicolectomy 5.3%) ( **Supplementary Table 3** ). Median polyp diameter was 2.5 cm (IQR 1.5–3.5). Polyps < 10 mm were found in 71 patients (11.4%) and no residual pathological evidence of a polyp was found in 23 patients (3.6%). The most commonly reported histologic characteristics were tubular adenoma (31.6%) and villous or tubulovillous adenoma (26.3%); 28.8% contained high-grade dysplasia or carcinoma in situ. Only 4.2% (N = 27) had histological evidence of invasive adenocarcinoma (submucosal invasion or more advanced).

### Cost analysis

Cost data were available for most patients (n = 5,826) with colectomies performed since 2003. Inflation-adjusted total costs for a given procedure and the associated hospitalization are presented in Table 3 . Median total costs were lower for procedures completed laparoscopically compared with those completed in open fashion. Patients who had any postoperative complication had significantly higher costs than those patients who did not ( *P* < 0.001 for both open and laparoscopic). Cost has steadily increased from $32,279.20 (SD $75,910.94) to $36,859.28 (SD $68,538.49).

**Table:** **Table 3** Cost of colectomy with and without complications.

**Procedure type**	**All cases** N = 5,826	**No complications** N = 4,678	**Any complications** N = 1,148	*P* value*
**Open colectomy**				
Median total hospital costs (IQR)	$23,120 ($16,467–33,209)	$21,752 ($15,764–29,150)	$33,033 ($20,782–56,191)	< 0.001
**Laparoscopic colectomy**				
Median total hospital costs (IQR)	$21,567 ($15,001–30,306)	$20,778 ($14,378–28,465)	$29,051 ($19,855–48,157)	< 0.001
Costs were adjusted for inflation to 2009 dollars. *Results of Chi-squared test comparing cost of colectomy with no complication versus any complications. IQR, interquartile range.				

## Discussion

Our study found that elective colectomies performed for treatment of benign colon polyps represented a large percentage of all colectomies performed annually, and importantly, that the percentage was increasing over time. This finding mirrors that reported by Peery et al. 24 of a non-veteran population and likely represents national trends ( Table 4 ). These findings are likely driven by a similar increase in the number of colonoscopy procedures completed. The percentage of colectomy procedures completed for cancer initially rose and then declined, illustrating the importance of colonoscopy in the screening and prevention (via polypectomy) of colon cancer 3 5 .

**Table:** **Table 4** Comparison of results from the current study with findings from Peery et al. and Bronzwaer et al.

	**Current study**	**Peery et al.**	**Bronzwaer et al.**
Population	US Veterans	US non-veterans (NSQIP)	Dutch
Year of study	2000–2015	2011–2014	2005–2015
Number of patients included	7,102	12,732 (only including patients with nonmalignant surgical resection)	915
Demographics			
Age	mean 66.0 years ± 8.8	largest age group 60–69 years: n = 4,191 (33%)	mean 67.4 years ± 10.0
Sex	M: n = 6,873 (96.8%)	M: n = 6,220 (49%)	M: n = 465 (50.8%)
	F: n = 229 (3.2%)	F: n = 6,504 (51%)	F: n = 450 (49.2%)
30-day mortality	1.2%	0.7%	1.4%
Complication rate	19.7%	14%	34.8%
Postoperative length of stay (days)	7.7 ± 7.0	5 days	6.0 (IQR 4.0–10.0)
Characteristics of lesions removed by surgical resection	n = 642	n = 1232	n = 915
Size	2.7 cm ± 1.7 cm	Unknown	3.5 cm (IQR 2.5–5.0)
Proximal location	n = 514 (80%)	Unknown	n = 616 (67.3%)
High-grade dysplasia or carcinoma in situ histology	n = 185 (28.8%)	Unknown	n = 305 (33.3%)
Adenomatous histology	n=390 (60.7%)	Unknown	n=697 (76.2%)
IQR, interquartile range.			

In our large cohort of 7,102 patients who underwent elective colectomy for a benign colon polyp, we found a 1.2% 30-day, and a nearly 20% composite postoperative morbidity rate in our surgical cohort ( Table 2 ). These findings are similar to those reported in the non-VA population 16 25 and are significantly higher than what has been reported for endoscopic resection 9 11 13 . Importantly, we found that 30-day mortality and overall morbidity and mortality have decreased with time; however, need for reoperation did not significantly change, even with increased utilization of laparoscopic colectomies.

In addition, we found that colectomy is expensive with a median cost of $21,567 (laparoscopic) to $23,120 (open) with a nearly $10,000 increase in cost with occurrence of any AE. Comparisons of postoperative morbidity and cost from our study suggest that attempted advanced endoscopic resection is likely to be cost-effective compared with surgical resection in the VA population, a finding mirrored in the non-VA population as well 18 20 . Thus, endoscopic management should be the goal in high-quality care of complex benign colon polyps.

Endoscopic resection is the preferred method over surgical resection for all benign colorectal neoplasms regardless of size, shape, or location with associated lower morbidity, mortality, and costs than surgical resection 15 17 20 26 27 . Recent studies have shown that advanced endoscopic techniques can be used to successfully manage complex polyps in up to 92% of cases 11 13 14 28 . Although not all endoscopists may perform these advanced techniques, referral to a provider specializing in endoscopic resection is now recommended 29 . Studies have shown that, among those referred for surgical resection, 58% to 74% had lesions that were amenable to endoscopic removal on repeat endoscopy, and that the cancer rate in the resected polyps was low 7 8 9 30 31 . The ASGE/ACG Colonoscopy Quality Indicators document encourages quality monitoring of management practices for benign colon lesions 32 . For example, they suggest measuring the fraction of non-pedunculated lesions < 2 cm that are referred for surgical resection and more recently, that sites review the pathology of all surgical colorectal resections to assess for inappropriate referral of benign lesions for surgery.

To better understand potential patient and polyp factors driving colectomy utilization, we analyzed pathology reports from a random subgroup of patients. Mean polyp size in this study was 2.8 cm, the predominance was right-sided in location, and only 28.8% of lesions contained high-grade dysplasia or carcinoma in situ histology. A predominance of proximal polyps was also reported by Bronzwaer and colleagues in a Dutch cohort ( Table 4 ) 33 . One possible explanation for preferential use of colectomy for proximal lesions may be a perceived increased risk of complications with endoscopic resection. Proximal colon location has been established as a risk factor for postprocedural bleeding 34 and perforation 35 .

At present, limited information explains the increased trend in surgical resection for benign polyps. A decline in training opportunities for EMR may have contributed to a reduced number of specialists proficient in advanced endoscopic resection. In a study of 415 providers, 40.4% felt their fellowship prepared them poorly for large polyp resection 36 . Advanced polypectomy has a steep learning curve, requiring deliberate practice and actionable feedback, ideally in a dedicated training environment. These findings raise important questions about endoscopy training: Are trainers placing sufficient emphasis on advanced resection? Do time and workload constraints limit opportunities for fellows to develop these skills? Does concern for patient safety discourage trainers from allowing hands-on experience? In such cases, simulation-based mastery learning (SBML) offers a potential solution. SBML is a rigorous education method that enables trainees to develop technical proficiency in a controlled, simulation-based environment, reducing reliance on patient-based training, especially for novices. By emphasizing competency and self-efficacy, SBML ensures that all trainees achieve proficiency before performing procedures in clinical practice.

There are several limitations of this study. First, we performed a retrospective analysis of an administrative dataset; therefore, there is potential for misclassification error. Notably, the VASQIP dataset is designed for quality improvement purposes with data entered by a trained nurse data manager, minimizing this risk. In addition, we excluded colectomies for colon cancer and emergent surgeries to ensure that colectomies were performed for benign polyps only. Although we were able to analyze colectomy outcomes and characteristics, we did not have the corresponding colonoscopy findings to fully assess risk factors for surgical resection versus endoscopic resection. We used pathology characteristics from resected specimens as a surrogate for polyp-related risk factors for surgery referral. However, we could not determine if prior endoscopic resection was attempted prior to surgical resection. Histology and size may have been worse on initial colonoscopy although the location of resection is less likely to be affected by this issue. We had limited information on size, shape, and histological features of the benign lesions. Cost estimates in our study likely underestimate the real costs associated with colectomy procedures because we did not include costs after discharge from the initial hospitalization. Finally, we note that we analyzed a veteran population with a higher burden of comorbidities based on the majority of patients with an ASA classification of III; therefore, anticipated outcomes of surgery would be worse than in a healthier population. However, we note that our findings are similar to those reported in a US non-VA population 16 as well as the Dutch population 33 . The study cohort was 96.8% men and 70.7% of White race. Generalizability of these findings may be limited by these demographics.

## Conclusions

Among a national cohort of US veterans, the increasing volume of colonoscopy procedures during the period from 2000 to 2015 resulted in a significant increase in the number of surgical colectomies performed for non-cancerous polyps. In the most recent full year of data, close to one-quarter of colectomies were performed for benign polyps. Surgical morbidity, mortality, and costs associated with these procedures are significant. Development of strategies to improve endoscopic management of benign large colon neoplasms is urgently needed.

## Supplementary Material

Supplementary Material Zusatzmaterial
