# Supplementary material for: Elective colectomy for treatment of benign colon polyps: National surgical trends, outcomes, and cost analysis
Source: Endosc Int Open. 2025 Oct 16;13:a26895839. doi: 10.1055/a-2689-5839 (PMC12550741; doi:10.1055/a-2689-5839)
Supplement: Supplementary file 1 — Supplementary Material [file 10-1055-a-2689-5839_26955774.pdf]

Supplementary Table 1 CPT and ICD-9 codes used for cohort identification.

	ICD-9/CPT code
**Laparoscopic colectomy**	44204, 44205
**Open colectomy**	44140, 44141, 44144, 44160
**Benign colon polyp**	211.3
**Colon cancer**	153.x, 154.x

CPT, Current Procedural Terminology; ICD, International Classification of Diseases.

Endosc Int Open | DOI 10.1055/a-2689-5839 | © The Author(s).

Supplementary Table 2 Surgical outcome variation over time.

	2000-2003 N = 984 (%)	2004-2007 N = 1741 (%)	2008-2011 N = 2315 (%)	2012-2015 N = 2065 (%)	*P* value
**Mortality** (30-day)	22 (2.2)	16 (0.9)	31 (1.3)	19 (0.9)	0.03
**Composite morbidity, mortality***	199 (20.2)	373 (21.4)	472 (20.4)	354 (17.1)	0.006
**Reoperation**	56 (5.7)	98 (5.6)	137 (5.9)	93 (4.5)	0.147
**Cardiac**					
MI	10 (1.0)	7 (0.4)	5 (0.2)	6 (0.3)	0.007
Cardiac arrest	11 (1.1)	13 (0.7)	14 (0.6)	13 (0.6)	0.159
Stroke/CVA	1 (0.1)	3 (0.2)	6 (0.2)	0 (0)	0.404
PE	2 (0.2)	13 (0.7)	15 (0.6)	10 (0.5)	0.725
DVT	4 (0.4)	8 (0.5)	12 (0.5)	10 (0.5)	0.752
**Pulmonary**					
> 48 hours on vent	15 (1.5)	42 (2.4)	43 (1.9)	32 (1.5)	0.415
PE	2 (0.2)	13 (0.7)	15 (0.6)	10 (0.5)	0.725
Pneumonia	27 (2.7)	59 (3.4)	63 (2.7)	46 (2.3)	0.119
Reintubation	22 (2.2)	55 (3.2)	49 (2.1)	44 (2.1)	0.233
**Infectious**					
Sepsis	12 (1.2)	46 (2.6)	88 (3.8)	43 (2.1)	0.225
SSI superficial	60 (6.1)	167 (9.6)	188 (8.1)	123 (6.0)	0.082
SSI deep	15 (1.5)	25 (1.4)	31 (1.3)	15 (0.7)	0.032
**Urinary tract/renal**					
UTI	23 (2.3)	59 (3.4)	63 (2.7)	41 (2.0)	0.126
ARF	4 (0.4)	11 (0.6)	18 (0.8)	10 (0.5)	0.886
Renal insufficiency	12 (1.2)	16 (0.9)	17 (0.7)	17 (0.8)	0.279
**Wound**					
Dehiscence	17 (1.7)	39 (2.2)	46 (2.0)	43 (2.1)	0.77

Results are reported as numbers of event and percent of total colectomies for benign polyps performed during the corresponding quartile.
*Any one or more VASQIP postoperative event.
ARF, acute renal failure; CVA, cerebrovascular accident; DVT, deep venous thrombosis; MI, myocardial infarction; PE, pulmonary embolism; SSI, superficial skin and soft tissue infection.

Endosc Int Open | DOI 10.1055/a-2689-5839 | © The Author(s).

Supplementary Table 3 Surgical pathology from randomly reviewed cases.

	**All reviewed colectomy cases**<br>N = 642 (%)
**Specimen**	
Ileocecectomy	26 (4.1)
Right hemicolectomy	514 (80.1)
Left hemicolectomy	34 (5.3)
Sigmoidectomy	32 (5.0)
Subtotal colectomy	7 (1.1)
Transverse/segmental colectomy	29 (4.5)
**Number of polyps**	
No residual	23 (3.6)
1	368 (57.3)
2	105 (16.4)
3	63 (9.8)
4+	4 (12.9)
**Polyp size, centimeters**<br>Mean (SD)	2.7 (1.7)
**Histology***	
Normal	24 (3.7)
Hyperplastic	6 (0.9)
Other	10 (1.6)
Sessile serrated adenoma	18 (2.8)
Tubular adenoma	203 (31.6)
Villous/tubulovillous adenoma	169 (26.3)
High grade dysplasia/carcinoma in situ	185 (28.8)
Submucosal invasion	9 (1.4)
Advanced cancer†	18 (2.8)

*For cases with greater than 1 polyp type reported, the most concerning polyp histology was used for categorization.
†Defined as stage 2 or greater colon cancer.
SD, standard deviation.

Endosc Int Open | DOI 10.1055/a-2689-5839 | © The Author(s).

Supplementary Figure 1 Use of laparoscopic colectomies for benign polyps has increased during our study period.

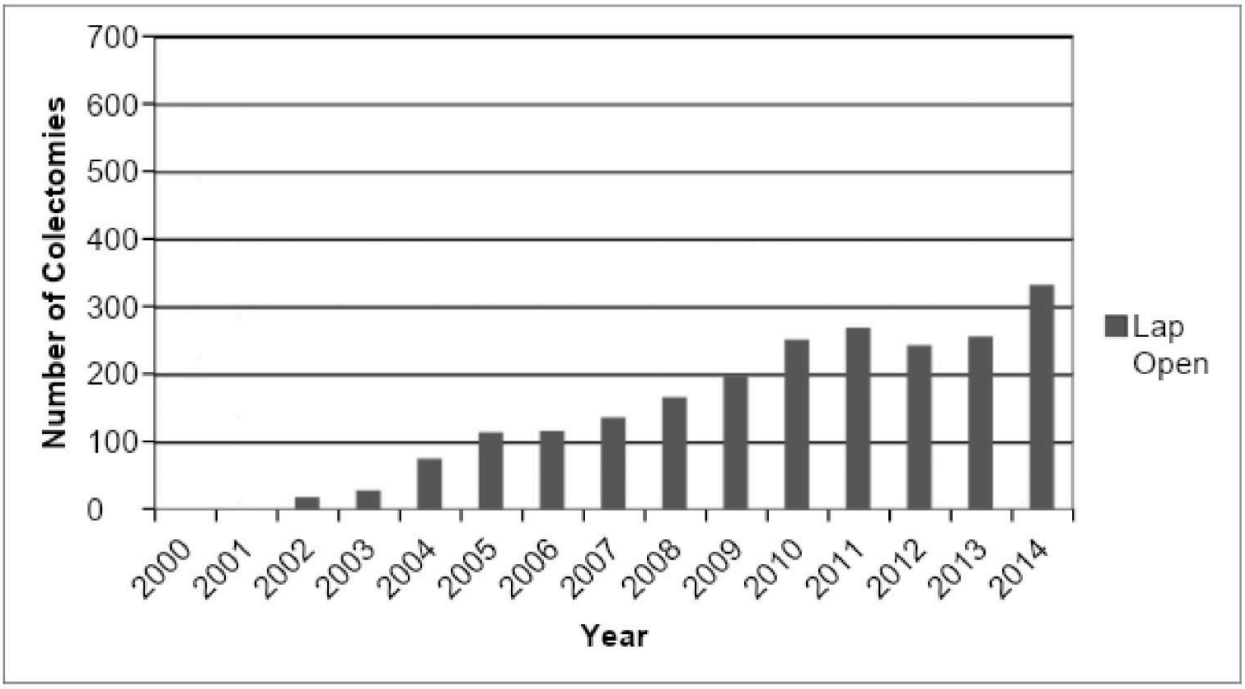

Endosc Int Open | DOI 10.1055/a-2689-5839 | © The Author(s).
